# Immunotoxicity and intestinal effects of nano- and microplastics: a review of the literature

**DOI:** 10.1186/s12989-020-00387-7

**Published:** 2020-11-12

**Authors:** Nell Hirt, Mathilde Body-Malapel

**Affiliations:** Univ. Lille, Inserm, CHU Lille, U1286- INFINITE - Institute for Translational Research in Inflammation, F-59000 Lille, France

**Keywords:** Microplastics, Nanoplastics, Intestinal, Microbiota, Inflammation, Immunotoxicity

## Abstract

**Background:**

Together with poor biodegradability and insufficient recycling, the massive production and use of plastics have led to widespread environmental contamination by nano- and microplastics. These particles accumulate across ecosystems - even in the most remote habitats - and are transferred through food chains, leading to inevitable human ingestion, that adds to the highest one due to food processes and packaging.

**Objective:**

The present review aimed at providing a comprehensive overview of current knowledge regarding the effects of nano- and microplastics on intestinal homeostasis.

**Methods:**

We conducted a literature search focused on the in vivo effects of nano- and microplastics on gut epithelium and microbiota, as well as on immune response.

**Results:**

Numerous animal studies have shown that exposure to nano- and microplastics leads to impairments in oxidative and inflammatory intestinal balance, and disruption of the gut’s epithelial permeability. Other notable effects of nano- and microplastic exposure include dysbiosis (changes in the gut microbiota) and immune cell toxicity. Moreover, microplastics contain additives, adsorb contaminants, and may promote the growth of bacterial pathogens on their surfaces: they are potential carriers of intestinal toxicants and pathogens that can potentially lead to further adverse effects.

**Conclusion:**

Despite the scarcity of reports directly relevant to human, this review brings together a growing body of evidence showing that nano- and microplastic exposure disturbs the gut microbiota and critical intestinal functions. Such effects may promote the development of chronic immune disorders. Further investigation of this threat to human health is warranted.

## Background

**Scheme 1:**
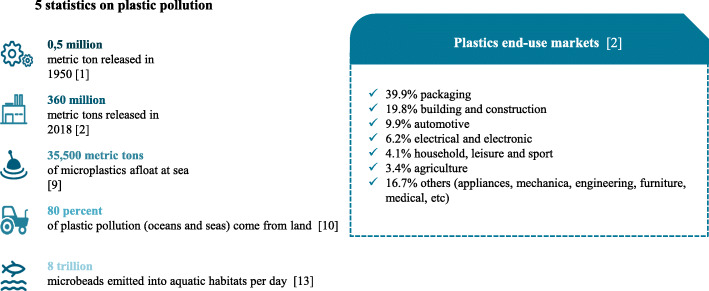
The omnipresence of plastics

The use of plastics has increased hugely over the past few decades. Indeed, the continuous production, use and consumption of plastics since the 1950s has created major environmental problems worldwide (Scheme 1). In 1960, half a million metric tons of plastics were released each year in the world [1]. This tonnage has since risen exponentially and has reached 359 million metric tons in 2018 [2]. In view of their low price and attractive physicochemical properties, plastics have become essential in every industry (packaging, construction, transport, etc). At present, it is almost impossible to find plastic-free goods. Plastic is used extensively in our everyday objects (packaging, cosmetics, household goods, electrical and electronic equipment, furniture, etc). Due to limited recycling and the lack of regulations limiting plastic waste, plastics (and especially nano- and microplastics) have contaminated aquatic, terrestrial and atmospheric environments worldwide. Plastics are present in our oceans, seas, rivers, and lakes, and have even reached the Arctic sea ice [3–5].

Microplastic pollution is ubiquitous in soil environments, including agricultural/farmland, greenhouse, home garden, coastal, industrial, and floodplain soils [6]. This pollution is due to the tremendous growth in plastic waste. In 2018, 25% of the 29.1 million metric tons of post-consumer plastic waste in Europe ended up in landfills [2]. Soil microplastics come from the unsustainable use and inappropriate waste management of plastics - especially those in packaging. Moreover, microplastics are released into the soil by agricultural processes [7]; the use of plastic mulches and the application of sewage sludge to fields are major sources of soil microplastics. In order to prevent microplastics from entering the aquatic environment, wastewater treatment plants remove microplastics from the wastewater but thus concentrate them in the sludge subsequently used as a fertilizer on agricultural soils [8].

In the marine environment, plastic debris can be found on the sea floor, surface and shoreline. Eriksen et al. estimated in 2014 that at least 5.25 trillion plastic particles including 35,500 metric tons of microplastics were floating at sea [9]. It has been estimated that 80% of the plastic pollution in the oceans and seas comes from land [10] and the estimated amount of land-based plastic debris entering the ocean is between 4.8 and 12.7 million metric tons per years [11]. Microplastics are detected in freshwater, including lakes, rivers, and groundwater. These particles come mainly from urban pollution but also from shipping, fisheries, tourism, oil and gas platforms, wastewater treatment plants, discharged personal health care products, textiles, and packaging [12]. Rochman et al. (2015) calculated that in 2015, 8 trillion microbeads per day were emitted into aquatic habitats in the United States [13].

Lastly, the atmosphere is a new recognized vehicle through which microplastics enter the wider environment [14, 15]. Microplastics have been measured in atmospheric fallout in both megacities [16, 17] and sparsely inhabited areas [18]. Suspended atmospheric microplastics have been also repeatedly detected in indoor air [19, 20].

The omnipresence of microplastics in the environment leads to human exposure largely by ingestion but also by inhalation and dermal contact [21]. This exposure is a cause of concern for potential long-term health hazards. Recent research has highlighted the possible adverse effects of nano- and microplastic exposure on intestinal homeostasis, gut microbiota and immune response. Here, we review this emerging field. Firstly, nano- and microplastics were briefly defined, then the pathways through which they can interact with the intestine and the immune system were described. Afterwards, studies of in vivo exposure to nano -and microplastics on the gut epithelium, the intestinal microbiota, and the immune system were detailed. The potential of microplastics as carriers of intestinal toxics and pathogens was emphasized. Lastly, current research perspectives and future needs were discussed.

## Definitions of MICROPLASTICS and NANOPLASTICS

**Scheme 2:**
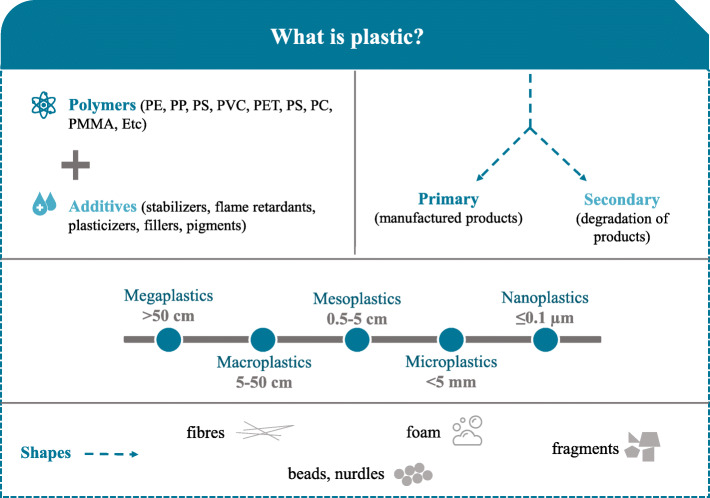
Definitions of plastics. PE: polyethylene, PP: polypropylene, PS: polystyrene, PVC: polyvinyl-chloride, PET: polyethylene Terephthalate, PC: polycarbonate, PMMA: poly methyl methacrylate, PU: polyurethane

The term “plastics” refers to any material containing a high polymer as an essential ingredient and has been discovered since the beginning of the twentieth century [22] (Scheme 2). Plastics consist of an assembly of polymers (polyethylene (PE), polypropylene (PP), polystyrene (PS), polyvinyl chloride (PVC), polyethylene terephthalate (PET), polycarbonate (PC), poly methyl methacrylate (PMMA), polyurethane (PU), etc) and additives (stabilizers, flame retardants, plasticizers, fillers, and pigments) that increase their performance [2, 23]. Plastic particles can be divided into two categories: primary particles are contained in manufactured products (personal health care products, etc) whereas secondary particles come from the degradation of products (packaging, clothes, etc). The degradation of plastics (photodegradation, oxidation, hydrolytic degradation, biodegradation) produces different forms and sizes of debris; nanoplastics (≤ 0.1 μm), microplastics (< 5 mm), mesoplastics (0.5–5 cm), macroplastics (5–50 cm), and megaplastics (> 50 cm) [24].

The term “microplastics” was used for the first time by Thompson et al. (2004) [25]. Microplastics are usually defined as “small ubiquitous plastic particles < 5mm in diameter”, but there is currently no consensus on the definition of microplastics [26, 27]. However, they can be characterized with regard to their size, shape and even by chemical composition. For example, microplastics can be subcategorized as a function of the shape as beads (in personal health care products), nurdles (pre-production of plastic beads), fibres (textile industry), foams (food industry, packaging) and fragments (degradation of plastic products), and also pellets, filaments, films, etc. Further categorization by shape has been suggested (e.g., cylindrical, disk, flat, ovoid, spheruloid, elongated, rounded, irregular, etc) [26] .

As is the case for microplastics, there is no internationally agreed definition of a nanoplastic. The European Food Safety Authority (EFSA) has defined nanoplastics as “a natural, incidental or manufactured material containing particles, in an unbound state or as an aggregate or as an agglomerate and where, for 50% or more of the particles in the number size distribution, one or more external dimensions is in the size range of 1 nm−100 nm”. However the upper size limit for nanoplastics is 100 nm in some definitions and 1000 nm in others [28]. Here, we adopted the value of 100 nm as the threshold between nanoplastics and microplastics. As is the case for microplastics, nanoplastics can originate from engineered material or can be produced by the fragmentation of larger plastic particles. Unfortunately, nanoplastics are difficult to detect. There are currently a number of methodological obstacles to the characterization and quantification of nano-sized particles [29].

## Human exposure to NANO- and MICROPLASTICS

### Human exposure to microplastics through ingestion

**Scheme 3:**
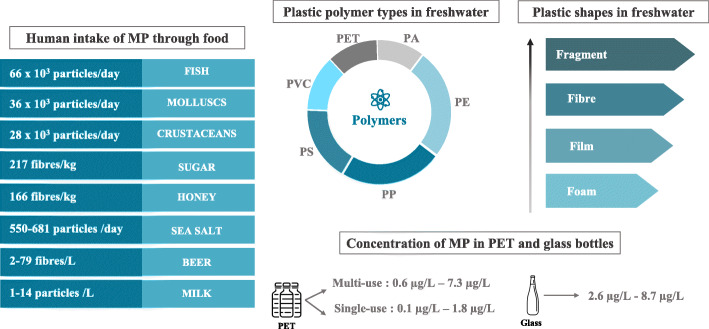
Human ingestion of microplastics. PE: polyethylene, PP: polypropylene, PS: polystyrene, PVC: polyvinyl chloride, PET: polyethylene terephthalate, PA: polyamide. Data on plastic polymers and shapes in freshwater are based on the number of studies reporting the presence of a particular polymer or shape of microplastic particles in freshwater. Adapted from Koelmans, Water Research 155 (2019) 410–422

The human ingestion of microplastics was revealed by the detection of microplastics in several dietary products (Scheme 3). Firstly, microplastics are ubiquitous in surface water, groundwater and wastewater [30]. Plastics are also found in drinking water and this issue has been reviewed recently [31, 32]. Koelmans et al. reported on the types of plastics found in freshwater (in decreasing order of frequency): fragments (35%), fibres (25%), films, foams, pellets, spheres, lines, beads, flakes, sheets, granules, paints, foils and nurdles. Overall, the polymers most frequently detected by researchers are PE ≈ PP > PS > PVC > PET, followed by polyamide (PA), acrylic or acrylic-related compounds, polyesters and PMMA. Despite the removal of microplastics by various water treatment processes, microplastics are also detected in tap water [33]. In Kosuth et al.’s study of 159 samples of tap water from all over the world, 81% contained microplastics; the mean concentration was 5.45 particles/L [34]. In an analysis of tap water samples in China, the lowest microplastic particle count measured was 440/L. Most of these particles were smaller than 50 μm fragments (followed by fibres and spheres) and composed mainly of PE and PP [35]. Microplastic particles were also detected in mineral water contained in both plastic bottles and glass bottles. The literature data on microplastics in mineral water were compiled recently [36]. The overall reported concentrations of microplastics were 0.6 μg/L [37] and 7.3 μg/L [38] in multi-use PET bottles, and 0.1 μg/L [37] and 1.8 μg/L [38] in single-use PET bottles. The concentrations in water in glass bottles were even higher (2.6 μg/L [37] and 8.7 μg/L [38]). The overall particle number ranged from 14 to 6290 particles/L [36]. Particles smaller than 5 μm accounted for approximately 96% of the total in PET bottles and 78% in glass bottles [39]. In a recent analysis of microplastics in Thailand, there were 140 particles/L in water in PET bottles, 52 particles/L in water in glass bottles, 81, 26 and 12 particles/L for the 6.5–20 μm, 20–50 μm, and > 50 μm diameter sizes [40]. The estimated maximum annual uptake by human adults is 458,000 microplastic particles for tap water and 3,569,000 microplastic particles for bottled water [32].

Given the presence of microplastics in the oceans, these particles are also detected in seafood products [41, 42]. Indeed, some of the 220 species found to ingest microplastic debris in natura (such as mussels, oysters, clams, common shrimps, etc) are of commercial importance for fisheries and aquaculture [43]. In Hantoro et al.’s review of studies of microplastics in seafood, it was estimated that the human intake can attain 66 × 10^3^, 28 × 10^3^ and 36 × 10^3^ particles/day through fish, crustacean, and mollusk consumption, respectively [44].

Furthermore, qualitative and quantitative measurements of microplastics have been reported for other food products, such as honey and sugar. In samples of these basic products collected in Europe (Germany, France, Italy, and Spain) and Mexico, the fibre content per kg was 166 for honey and 217 for sugar [45].

Microplastic contamination has also been detected in sea salt originating from various countries worldwide [34, 46–49], and these data have been reviewed by Toussaint et al. [26]. For example, 550–681 particles/kg were detected in sea salt samples collected across China, [46]. The majority of the particles (55%) measured less than 200 μm in diameter. Fragments and fibres were more prevalent than pellets and sheets. The most common microplastics were PET, followed by PE and cellophane.

Furthermore, Liebezeit et al. analyzed the content of microplastics in German beers [50]. Microplastic contamination was found in all cases, with counts ranging from 2 to 79 fibres/L, from 12 to 109 fragments/L and from 2 to 66 granules/L. The relative contributions ranged from 5 to 71% for granular material, from 14 to 87% for fragments and from 3 to 57% for fibres.

Microplastics have been also detected in cow milk samples for adults and children. All samples contained microplastic particles, with differences in the amounts (1 to 14 particles/L) [51]. Of the total detected microplastics, 97.5% were fibres and 2.5% were fragments: microplastics < 0.5 mm were dominant (40%) followed by the sizes 0.5–1 mm (28%) and 1–2 mm (25%).

Lastly, many researchers have attempted to estimate the yearly human exposure to microplastics, However, interstudy differences in the types of plastic and the experimental methods mean that these estimations vary markedly [26]. In such a context, another way to estimate human contamination is probably to measure the amount of plastic in human feces. This is what Schwabl et al. did in a recent study of stools samples from 8 healthy volunteers: the mean number microplastic particles (from 50 to 500 μm in size) was 20 per 10 g. Nine types of plastic were detected, with PP and PET being the most abundant. Based on these results and an average production of 128 g of feces per day per person, the researchers estimate that the annual discharge of microplastic particles in the feces (reflecting at least in part the equivalent human body exposure) was over 90,000 [39].

### Other routes of human exposure to nano- and microplastics

Human exposure to microplastics also occurs through inhalation, because microplastics are present in the indoor and outdoor air [52]. The sources of airborne microplastics have been reviewed recently [14, 15]. Synthetic textiles, the erosion of synthetic rubber tires, and city dust are thought to be the most important sources of airborne microplastics. Fibres were the dominant shape, and PP, PE, PS and PET were the dominant polymer components of microplastics in atmospheric fallout. Individual human exposure by inhalation has been estimated at 26 to 130 airborne microplastic particles a day [14].

Lastly, microplastics reach humans by dermal contact. Microplastic beads are included in the composition of facial cleansers, facial scrubs, and toothpaste, where they are used as exfoliators for the skin and teeth [53]. A recent Chinese study has identified personal care products containing microplastics. Overall, 7.1% of facial cleansers contained microplastics, with a mean ± standard deviation content of 25.04 ± 10.69 mg microplastics/g and mean size of 313 ± 130 μm. The majority of these microplastics were made of PE [54]. Microplastic beads are also used to regulate the viscosity of films, condition the skin, and stabilize emulsions: they are included into a wide range of products, such as soaps, shampoos, deodorants, wrinkle creams, moisturizers, shaving creams, sunscreen lotions, facial masks, lipsticks, eye shadows, and children’s bubble bath [55]*.* The glitters which are used in significant volumes in make-up (and also craft activities and textile products) are usually PET-containing plastics [56]*.* Moreover, the microbeads used in consumer products (such as scrubs and shampoos) are processed by mechanical means, which may lead to their fragmentation into potentially more hazardous nanoplastics. The presence of nanoplastics has been confirmed in personal care products containing PE microbeads [57].

## Relationships between NANO- and MICROPLASTICS, the intestinal mucosa, and the immune system

Many studies of various species have shown that ingested microplastics accumulate in the gut of various species [58–61]. After a five-day oral course of 60 nm PS nanoparticles in rats, approximatively 10% of the dose was found in the gastrointestinal tract [62]. Not much is known on the distribution of nano- and microplastics after ingestion. Based on in vitro and in vivo data, knowledge on the uptake of nano- and microplastics has been reviewed by the European Food Safety Authority [63]. Microplastics with a greatest dimension > 150 μm are not absorbed, they remain bound to the intestinal mucus layer and come into direct contact with the apical part of intestinal epithelial cells. This may lead to inflammation of the gut and local effects on the immune system. The smaller particles (greatest dimension < 150 μm) can cross the mucus barrier. Indeed, several mechanisms result in the size-dependent uptake of nano- and microparticles: (i) endocytosis through enterocytes, (ii) transcytosis through microfold cells (also referred to as M-cells, a specific subset of intestinal epithelial cells in gut-associated lymphoid tissue), (iii) persorption (namely the passage through “gaps” at the villous tip, following the loss of enterocytes), and (iv) paracellular uptake [64]. Uptake of microparticles by endocytosis, transcytosis and paracellular diffusion between enterocytes has been observed in rodents without any disruption of the intestinal barrier [65, 66]. Peyer’s patches have a high proportion of M-cells and constitute the main site of microplastic absorption [67]. The intestinal uptake of microparticles is not very efficient: in one study, only 0.3% orally administrated latex particles (greatest dimension: 2 μm) crossed the epithelium [68]. Despite this low level, intestinal absorption of particles may lead to systemic toxicologically relevant exposure. The small size of nanoplastics allows them to penetrate deeply into organs. Data from animal studies have shown that once absorbed, nanoplastics can distribute to the liver, spleen, heart, lungs, thymus, reproductive organs, kidney, and even the brain (i.e. they cross the blood–brain barrier [69]) [63].

It must also be borne in mind that airborne microplastics may also have an impact on the digestive tract and the immune system. It is known that among airborne particles, the smallest particles (i.e. the inhalable fraction) are absorbed via the pulmonary epithelium [70, 71]. They reach the systemic circulation and exert an immune effect on the so called gut-lung axis [72]. A proportion of the larger particles (the extrathoracic fraction) is transported to the gastrointestinal tract by mucociliary clearance, where it undergoes the fate of ingested particles. Hence, depending of the particle size, both ingested and inhaled plastics are able to interact with intestinal tissues, reach the bloodstream and (potentially) dysregulate the immune response.

## Effects of exposure to NANO- and MICROPLASTICS on the gut epithelium

Several in vivo studies have explored the effects of nano- and microplastic exposure on the gut epithelium (Table 1). Intestinal impact has been demonstrated in invertebrates. In *C elegans* nematode, exposure to a cocktail of microplastics (PE, PP, PVC and PS particles, from 0.1 to 70 μm in size and at a concentration of 5 mg m^− 2^, for 2 days) was associated with a significant reducttion in intestinal calcium levels and elevated intestinal expression of the enzyme glutathione S-transferase 4, suggesting that intestinal oxidative damage is a key mechanism in microplastic toxicity [74]. In *Artemia parthenogenetica* zooplankton larvae, exposure to low levels of PS microspheres (10 μm, 10 particles/mL, over 14 days) was associated with the progression of cellular deformations and the enterocyte decomposition [75]. In the mussel *Mytilus spp*, exposure to PS microbeads (2 and 6 μm, 32 μg/L, for 7 days) also resulted in alterations in the oxidative balance in the digestive gland (a reduction in catalase and glutathione reductase activities, and in lipid peroxidation) [76]. Another study of exposure of mussels to PS beads but on the nano scale level revealed that PS nanoplastics (110 nm, 0.05 to 50 mg/L, for 96 h) increased *Hsp70* mRNA levels, total oxidant status, total antioxidant capacity, and lipid peroxidation in the digestive gland [77]. Exposure of mussels to a mixture of PE and PS microbeads (< 400 μm, for 10 days) was associated with lower glutathione S transferase and superoxide dismutase activities (at 100 μg/L) and greater superoxide dismutase and catalase activities (at 0.008 and 10 μg/L) [78].

**Table 1:** Overview of in vivo studies of the effects of nano- and microplastic exposure on the gut epithelium. *This has been calculated by the authors based on Bachmanov AA et al. *Behav Genet* 2002 [73]

Reference	Nano-microplastics	Dosage	Duration of exposure	Route of exposure	Species	Observed effects related to the gut epithelium
Invertebrates						
Lei et al., Sci Total Environ. 2018 [74]	Polyamides, polyethylene, polypropylene, polyvinyl chloride and polystyrene 0.1 to 70 μm	5 mg m^− 2^	2 days	Added to the nematode’s growth medium	Nematode *(Caenorhabditis elegans)*	↓ intestinal calcium levels ↑ glutathione S-transferase 4 enzyme expression
Wang et al., Chemosphere 2019 [75]	Polystyrene 10 μm	10 particles/mL	14 days	Culture medium	Zooplankton *(Artemia parthenogenetica)* larvae	Histological deformation and destructuring of the intestinal epithelium
Paul-Pont et al., Environmental Pollution 2016 [76]	Polystyrene microbeads (2 and 6 μm)	32 μg/L	7 days	Supplied with *Chaetoceros mueller* algae as a food source	Mussel *(Mytiulus spp)*	In digestive gland ↓ catalase activity ↓ glutathione reductase activity ↓ lipid peroxidation
Brandts et al., Sci Total Environ 2018 [77]	**Polystyrene** **110 nm**	0.05 to 50 mg/L	96 h	Tank water	Mussel *(Mytiulus galloprovincialis)*	In digestive gland ↑ *Hsp70* mRNA levels, total oxidant status, total antioxidant capacity, and lipid peroxidation
Revel et al., Frontiers in Environmental Science 2019 [78]	Commercial polyethylene and polystyrene mixture (< 400 μm)	0.008, 10, 100 μg/l	10 days	Tank water	Mussel (*Mytilus spp.)*	↓glutathione S transferase and superoxide dismutase activities (at 100 μg/L) ↑superoxide dismutase and catalase activities (at 0.008 and 10 μg/L)
Vertebrates						
Asmoniate et al., Environ. Sci.Technol. 2018 [79]	Polystyrene 100–400 μm	10 mg /fish/day	4 weeks	Food	Rainbow trout *(Oncorhynchus mykiss)*	No variations in paracellular permeability, intestinal tight junction and cytokines mRNA expression, or ion transport
Huang et al., Sc Total Environ. 2020 [80]	Polystyrene; 32–40 μm	100 and 1000 μg/L	28 days	Tank water	Juvenile guppy (*Poecilia reticulata*)	↓ digestive enzymes activity ↑ goblet cells secretion ↑ gut secretion of TNFα, IFNγ and IL6
Ahrendt et al., Mar Pollut Bull 2020 [81]	Poly(styrene-co-divinylbenzene) 8 μm	0.02 and 0.2 g/g food	Once a day for45 days	Diet	Juvenile intertidal fish *(Girella laevifrons)*	Dose-dependent whole intestine histological damage: leukocyte infiltration, hyperemia, and crypt and villus cell loss
Gu et al., J. Hazard. Mater. 2020 [82]	**Polystyrene** **100 nm**	10^4^ and 10^6^ particles/L	14 days	Tank water	Juvenile large yellow croaker	↓ digestive enzymes activity (lipase, trypsin, and lysozyme)
Kang et al., J Hazard. Mater. 2020 [83]	Polystyrene 50 nm (NP) and 45 μm (MP)	2.5 μg/mL	14 days	Artificial sea water	Medaka (*Oryzias. melastigma*)	↑ mucus secretion (NP and MP) No variation of villus length and width (NP and MP) ↑ gut D-lactate levels (MP) ↑ gut diamine oxidase levels (NP and MP) Gut oxidative stress: NP: ↓ ROS, ↑ SOD, ↑ CAT, ↑ GST MP: ↑ ROS, ↓ SOD, ↓ CAT, no variation of GST
Jin et al., Environ. Pollut. 2018 [84]	Polystyrene 0,5 and 50 μm	1000 mg/L	14 days	Tank water	Zebrafish *(Danio rerio)*	0.5 μm beads: ↑ gut mRNA and protein levels of IL1α, IL1β and IFN 50 μm beads: no differences
Qiao, Sheng, et al., Sci.Total Environ. 2019 [85]	Polystyrene 5 μm	50 & 500 μg/L	21 days	Tank water	Zebrafish *(Danio rerio)*	↑ catalase and superoxide dismutase activities ↓gut D-lactate content
Gu et al., Environ. Sci. Technol. 2020 [86]	Polystyrene **100 nm**, 5 μm, 200 μm	500 μg/L	21 days	Tank water	Zebrafish *(Danio rerio)*	↑ intestinal level of TLR2 protein (100 nm, 200 μm) ↑ mucus secretion (100 nm) Significant transcriptome variations: specific of the NP/MP type, and specific of the intestinal cell population (enterocytes, secretory cells, M1and M2 macrophages, T and B cells)
Qiao, Deng, et al., Chemosphere. 2019 [59]	Polystyrene Beads 15 μm Fragments 4-40 μm Polypropylene Fibres 20–200 μm	10 μg/L	21 days	Tank water	Zebrafish *(Danio rerio)*	↓ mucus secretion (fibres) ↑ superoxide dismutase activity ↓ D-lactate levels ↑ Il1α levels (fragments and fibres)
Peda et al., Environ Pollut 2016 [87]	Polyvinyl chloride < 0.3 mm	1%w/w in food	90 days	Food	European sea bass *(Dicentrarchus labrax L)*	Histopathological alterations in the distal intestine (edema, villus desquamation, detached epithelium, and loss of epithelial structure)
Espinosa et al., Fish Shellfish Immunol. 2017 [88]	Polyvinyl chloride Polyethylene 40–150 μm	100 and 500 mg/kg of diet	3 weeks	Food	Gilthead seabream *(Sparus aurata)*	PVC 500 mg/kg: ↑ goblet cells count, villus thickness, and expression of intestinal nuclear factor E2-related factor 2 *Nrf2* PE 100 and 500 mg/kg: ↓ goblet cell count and villus height
Jabeen et al., Chemosphere 2018 [89]	Ethylene vinyl acetate 0.7-5 mm fibres	55–76 fibres per fish/day	3 days a week for 6 weeks	Food	Goldfish *(Carassius auratus)*	Histologically documented inflammatory infiltration and breakage of epithelium in the proximal and distal intestine
Limonta et al., Sci rep 2019 [90]	Irregularly shaped high density polyethylene and polystyrene particles	100 and 1000 μg/L	20 days	Food	Zebrafish (*Danio rerio)*	In the intestinal epithelium: epithelial detachment, ↑ neutrophils count ↓ goblet cell count
Lu et al., Sci.Total Environ. 2018 [91]	Polystyrene 0.5 and 50 μm	100 and 1000 μg/L ~ 26 and 266 μg/kg bw/day*	5 weeks	Drinking water	ICR mice *(Mus musculus)*	↓ mucus secretion ↓ *Muc1* transcript levels ↓ *Klf4* transcript levels (1000 μg/L only)
Jin et al., Sci.Total Environ. 2019 [60]	Polystyrene 5 μm	100 and 1000 μg/L ~ 26 and 266 μg/kg bw/day*	6 weeks	Drinking water	ICR mice *(Mus musculus)*	↓ *Muc1* and *Klf4* transcript levels ↓ *Cftr, Nkcc1* and *Nhe3* transcription in the colon ↓ *Ano1, Cftr, Slc26a6, Nkcc1*and *Nhe3* transcription in the ileum
Stock et am. Arch Toxicol 2019 [92]	Polystyrene 1,4, 10 μm	1.25, 25 and 34 mg/kg bw	28 days	Oral gavage	C57BL/6NTac mice *(Mus musculus)*	Absence of histologically detectable lesions or inflammatory responses.
Li et al., Chemosphere. 2020 [93]	Polyethylene 10–150 μm	2–20-200 μg/g Food ~ 0.0004, 0.004 and 0.04 μg/kg bw/day*	5 weeks	Food	C57BL/6 mice *(Mus musculus)*	In both colon and duodenum (200 μg/g only) ↑ histological score ↑ TLR4, AP-1 and IRF5 protein expression
Deng et al., Environment International 2020 [94]	Polyethylene 45–53 μm	100 mg/kg/day 5.25 10^4^ particles/day	30 days	Gavage	CD-1 mice (Mus musculus)	↑ serum D-Lactate levels No variation serum diamine oxidase activity ↓ gut transcript levels of *Cyp1a2*, *Cyp1a5*, *H2BMb2*, *H2Eb1*, *Aldh8a1*, *Scarb1* ↑ gut transcript levels of *Rdh16*, *Gm8909*

The intestinal effects of oral exposure to microplastics have been also demonstrated in vertebrates, namely in aquatic vertebrates and in mice. In aquatic vertebrates, several studies have explored the effects of PS. In the rainbow trout, exposure to PS beads (100–400 μm, 10 mg/fish/day for 4 weeks) was not associated with any variation in paracellular permeability, ion transport, and intestinal tight junction and cytokines mRNA expression [79]. In juvenile guppies, exposure to PS microbeads (32–40 μm, 100 and 1000 μg/L for 28 days) decreased digestive enzymes activity, induced goblet cells enlargement, and gut TNF α (Tumor Necrosis Factor α), IFN γ (Interferon γ) and IL-6 (Interleukin-6) secretion [80]. In juvenile intertidal fish *Girella laevifrons*, histological analyses of the intestine showed that the exposure of microplastics (8 μm poly (styrene-co-divinylbenzene), 0.02 and 0.2 g/g of food, once a week for 45 days) led to leukocyte infiltration, hyperemia, and the loss of villi and crypt cells [81]. In the juvenile large yellow croaker *Larimichthys crocea*, exposure to PS nanospheres (100 nm, 10^4^ and 10^6^ particles/L, for 14 days) was associated with lower activities of several digestive enzymes (lipase, trypsin, and lysozyme) [82]. Another study assessed the effects of both nano-sized (50 nm) and micro-sized (45 μm) PS particles (2.5 μg/mL, for 14 days) in marine medaka *Oryzias melastigma*: some modifications were shared between both sizes of particles, such as the increase of mucus secretion and gut diamine oxidase levels. On the contrary, gut levels of oxidative stress enzymes were modified in opposite ways between nano-sized and micro-sized particles [83].

Moreover, several studies have explored the intestinal effects of PS exposure on zebrafish. In one study, exposure to PS beads (0.5 μm, 1000 mg/L, for 14 days) increased both gut mRNA and protein levels of the major proinflammatory cytokines IL1α, IL1β and IFN [84]. At a 10-fold-lower dosage or after exposure to larger PS beads (50 μm) under the same experimental conditions, these upregulations were not observed [84]. In another study, exposure to PS beads (5 μm, 50 and 500 μg/L, for 21 days) enhanced catalase and superoxide dismutase activities, reflecting an excessive oxidative stress. The exposure was also associated with lower levels of the antioxidant enzyme diamine oxidase and of D-lactate, a fermentation metabolite produced by various bacteria in the gut, which reflected an increase in intestinal permeability [85]. Lastly, single-cell RNA sequencing was used to determine the intestine-specific effects of PS nano-and microplastic beads (100 nm, 5 and 200 μm, 500 μg/L, for 21 days) in the zebrafish [86]. The transcriptome profiles revealed dysfunctions of intestinal cell populations, e.g. immune response of enterocytes, phagocytes and lymphocytes, detoxification/antioxidant capacity of enterocytes, cell chemotaxis of secretory cells. These effects were dependent on the particle size and specific of the intestinal cell population. They were associated with increased number of pathogenic intestinal bacteria.

The influence of the microplastic’s shapes on their gut toxicity has been analyzed in the zebrafish [59]. Exposure to PS beads, PS fragments or PP fibres (10 μg/L, for 21 days) decreased intestinal D-lactate levels. Microplastic fibres also induced a steep decline in the volume of mucus in the gut. Microplastic fibres and fragments caused intestinal inflammation, as characterized by the significant increase in the level of Il1α in the gut. Microplastic fragments, fibres, and beads also enhanced the activity of superoxide dismutase [59]. Hence, a growing body of evidence suggests that PS can induce oxidative stress and epithelial disruption in the intestine of aquatic species.

The effects of other types or shapes of microplastic have been also assessed in aquatic vertebrates. In the European sea bass *Dicentrarchus labrax* L, exposure to PVC (< 0.3 mm, 1%w/w in food) for 90 days induced histological alterations in the intestine (mainly in the distal part) [87], and contamination of the diet with 500 mg/kg PVC for three weeks (40 to 150 μm) increased the goblet cells number, the villus thickness, and expression of intestinal nuclear factor E2-related factor 2 *Nrf2*. Contamination with PE at the same dosage and a lower one (100 mg/kg) decreased the goblet cell number and the villus height [88]. In the goldfish *Carassius auratus*, oral exposure to ethylene vinyl acetate fibres (0.7–5 mm fibres, 55–76 fibres per fish per day, three days a week for 6 weeks) induced histologically confirmed inflammatory infiltration and breakage of epithelium in the proximal and distal intestine [89]. Epithelial detachment, an increase in the neutrophil count, and a decrease in the goblet cell count were observed in the intestine of zebrafish exposed for 20 days to irregularly shaped high-density PE and PS particles (100 and 1000 μg/L) [90].

Furthermore, evidence of the microplastics’ intestinal toxicity is now emerging in mammals. Three studies of the effects of PS have been conducted in the mouse. Exposure to microspheres (0.5 and 50 μm in diameter, 100 and 1000 μg/L, for 5 weeks) decreased the mucus secretion and the transcript levels of a major gene related to mucin expression, mucin 1 (*Muc1*) in colon [91]. Similarly, exposure of mice to PS microspheres microplastics (5 μm, 100 and 1000 μg/L, for 6 weeks) significantly decreased the secretion of mucus and the transcript levels of two genes related to mucus secretion, Mucin1 (*Muc1*) and Kruppel like factor 4 (*Klf4*) in the gut. After exposure to 1000 μg/L PS microspheres, there was significant down-regulation of the genes related to ion transport, such as cystic fibrosis transmembrane conductance regulator (*Cftr*), Na-K-2Cl cotransporter 1 (*Nkcc1*) and Na+/H+ exchanger 3 (*Nhe3*) in the colon, and anoctamin 1 (*Ano1*), *Cftr*, solute carrier family 26 member 6 (*Slc26a6*), *Nkcc1*and *Nhe3* in the ileum [60]. In contrast, no evidence of inflammation (from the duodenum to the colon) was found in another study of mice exposed to PS microplastics (1, 4 and 10 μm, 1.25, 25 and 34 mg/kg bodyweight by oral gavage, three times per week for 28 days); however, the mouse had a different genetic background, the exposure schedule differed, and a much higher dosage was used [92].

Two last studies in the mouse assessed the effects of another type of microplastic: mice were exposed to different amounts of polyethylene microplastics (10–150 μm, 2, 20, 200 μg/g of food, for 5 weeks). The mice showed clear signs of histological inflammation in the colon and duodenum, and expressed higher protein levels of the innate immune receptor toll-like receptor 4 (TLR4), the proinflammatory transcription factor activator protein 1 AP-1 (also known as c-Jun) and interferon regulatory factor 5 (IRF5) [93]. Another study of exposure of mice to polyethylene microplastiques (45–53 μm, 100 mg/kg/day by gavage, for 30 days) revealed impairments of intestinal permeability (increase of serum D-Lactate levels) and gene expression (decrease of gut transcript levels of *Cyp1a2* (cytochrome P450, family 1, subfamily a, polypeptide 2), *Cyp1a5* (cytochrome P450, family 1, subfamily a, polypeptide, *H2-DMb2* (histocompatibility 2, class II, locus Mb2), *H2-Eb1* (histocompatibility 2, class II antigen E beta), *Aldh8a1* (aldehyde dehydrogenase 8 family, member A1), *Scarb1* (scavenger receptor class B, member 1) and increase of gut transcript levels of *Rdh16* (retinol dehydrogenase 16), *Gm8909*) [94].

## Effects of NANO- and MICROPLASTIC exposure on the gut MICROBIOTA

The variations in the intestinal microbiota following in vivo exposure to microplastics have been investigated in several contexts. In the sea bass, an analysis of the gut microflora’s composition (using denaturing gradient gel electrophoresis fingerprinting) failed to detect shifts in the composition of the bacterial community after 90 days of exposure to native and weathered PVC [95]. In the shrimp, the size, granularity, and viability of gut microbial cells were greater in a group exposed for 7 days to PS nanoparticles (44 nm, 50 μg/mL of tank seawater) than in a control group. The cell viability of the gut microbiota was still increased after 2 and 3 weeks of exposure [96].

Other studies have used metagenomic techniques to explore changes in the gut microbiota (Table 2). In the common springtail *Folsomia candida* exposed to PVC microspheres (80 to 250 μm, 1 μg/kg dry soil for 56 days), the gut microbial diversity was significantly higher and its composition differed significantly, with fewer *Bacteroidetes* and more *Firmicutes* [97]. Exposure of *Folsomia candida* to PE (< 500 μm, at concentrations of 0.5% dry weight in the soil, for 28 days), significantly altered the microbial communities and decreased bacterial diversity in the springtail gut [98]. In the crab *Eriocheir sinensis*, 21 days of exposure to PS microspheres (5 μm, 40 mg/L) decreased the relative abundance of the *Firmicutes* and *Bacteroidetes*, and increased the relative abundance of the *Fusobacteria* and *Proteobacteria* [99].

**Table 2:** Overview of in vivo studies of the effects of nano- and microplastic exposure on the gut microbiota (metagenomic analyses). *This has been calculated by the authors based on Bachmanov AA et al. *Behav Genet* 2002 [73]

Reference	Nano-micro plastics	Dosage	Duration of exposure	Route of exposure	Species	Effects on bacterial diversity	Effects on bacterial phyla composition	Effects on bacterial genera composition
**Invertebrates**								
Zhu et al., Soil Biology and Biochemistry 2018 [97]	Polyvinyl chloride particles 80 to 250 μm	1 μg/kg dry soil	56 days	Soil	Springtail *(Folsomia candida)*	Alpha diversity ↑ (*p* < 0.01)	*↓ Bacteroidetes* *↑ Firmicutes*	(Family level: ↑ *Bacillaceae*)
Ju et al., Environ. Pollut. 2019 [98]	Polyethylene < 500 μm	0.5% dry weight in soil	28 days	Soil	Springtail, (*Folsomia candida)*	Chao1 diversity index ↓ Phylogenetic diversity whole-tree index↓	No data	↓*Wolbachia* ↑*Bradyrhizobiaceae*, *Ensifer* and *Stenotrophomonas*
Liu et al., Sci.Total Environ. 2019 [99]	Polystyrene 5 μm	40 mg/L	21 days	Tank water	Crab (*Eriocheir sinensis)*	Shannon diversity index: ↓	*↑ Cyanobacteria, Chloroflexi,* *Fusobacteria* and *Proteobacteria* *↓ Nitrospirae, Firmicutes,* *and Bacteroidetes*	25 significantly different
**Vertebrates**								
Huang et al., Sc Total Environ. 2020 [80]	Polystyrene; 32–40 μm	100 and 1000 μg/L	28 days	Tank water	Juvenile guppy (*Poecilia reticulata*)	Shannon diversity index: ↓ Simpson diversity index↑	*↑ Proteobacteria* *↓ Actinobacteria*	↑ *Gemmobacter* and *Rhodobacter*
Wan et al., Chemosphere 2019 [100]	Polystyrene 5 and 50 μm	1000 μg/L	7 days	Tank water	Larval zebrafish *(Danio rerio)*	Chao1 diversity index: ↓ (5 μm)	No significant variation	5 μm: ↓*Sphaerotilus*, *Haliangium* and *Leptothrix* ↑*Methyloversatilis*, *Polynucleobacter*, *Legionella* and *Ottowia* 50 μm: ↓ *Pseudomonas* ↑ *Flectobacillus* and *Methylophilus* 5 and 50 μm: ↓ *Methylobacterium*
Gu et al., J. Hazard. Mater. 2020 [82]	**Polystyrene** **100 nm**	5.5 × 10^− 12^ mg/L	14 days	Tank water	Large yellow Croaker (*Larimichthys croceus*)	Chao1 diversity index ↓ No variation of Shannon diversity index	↑ *Bacteroidetes, Firmicutes* *↓ Proteobacteria*	↑ *Lactobacillus, Parabacteroides, Alistipes*
Kang et al. J Hazard. Mater. 2020 [83]	Polystyrene **50 nm (NP**) and 45 μm (MP)	2.5 μg/mL	14 days	Artificial sea water	Medaka (*Oryzias. melastigma*)	Alpha diversity ↑ (MP only)	*↓ Bacteroidetes* (NP and MP),	NP and MP: *↓ Vicingus, Shewanella* ↑ *Lewinella, Pseudomonas, Thalassospira, Parahaliea*.
Jin et al., Environ. Pollut. 2018 [84]	Polystyrene 0,5 and 50 μm	1000 mg/L	14 days	Tank water	Zebrafish *(Danio rerio)*	Shannon diversity index: ↑	*↑ Firmicutes,* *↓ γ-Proteobacteria*	29 significantly different
Qiao, Sheng, et al., Sci.Total Environ. 2019 [85]	Polystyrene 5 μm	50–500 μg/L	21 days	Tank water	Zebrafish *(Danio rerio)*	Shannon diversity index: ↓ 17–29% in the 50 and 500 μg/L MP groups respectively (*p* < 0.05)	*↑ Fusobacteria* *↓ Proteobacteria*	(Family level: ↑ 12 ↓ 13)
Qiao, Deng, et al., Chemosphere. 2019 [59]	Polystyrene fibre 20-100 μm	10 μg/L	21 days	Tank water	Zebrafish *(Danio rerio)*	Abundance coverage-based estimator: ↑ 107.5% in the fibre-MP group (p < 0.05) Simpson’s diversity index: ↓ 45.7% in the fibre-MP group (p < 0.05)	*↑ Proteobacteria,* *↓ Actinobacteria,*	*↑ Gordonia* *↓ Aeromonas, Pseudomonas*
Jin et al., Sci.Total Environ. 2019 [60]	Polystyrene 5 μm	1000 μg/L ~ 266 μg/kg bw/day*	6 weeks	Drinking water	ICR Mice (*Mus musculus*)	Phylogenetic diversity whole-tree index ↓	↓ *α−Proteobactria* *γ-Proteobacteria*	*↓ Parabacteroides, Prevotella,* *Dehalobacterium, Turicibacter,* *Bifidobacterium, Phascolarctobacterium,* *Lachnospira, Haemophilus, Adlercreutzia,* *Megamonas, Blautia, Dialister* and *Veillonella* *↑ Coprococcus* and *Anaeroplasma*
Lu et al., Sci.Total Environ. 2018 [91]	Polystyrene 0.5 and 50 μm	1000 μg/L ~ 266 μg/kg bw/day*	5 weeks	Drinking water	ICR Mice (*Mus musculus*)	No data	*↓ Firmicutes,* *α-Proteobacteria,* *Actinobacteria*	*↓ Oscillospira and Anaerostipes* *↑ Parabacteroides, Prevotella,* *Dehalobacterium, Ruminococcus, Bilophila,* *Bifidobacterium, Adlercreutzia, Plesiomona, Halomonas* and *Acinetobacter* (after both 0.5 and 50 μm polystyrene MP exposure)
Luo et al., Environ. Sci. Technol 2019 [101]	Pristine polystyrene microspheres 5 μm	1000 μg/L ~ 266 μg/kg bw/day*	Gestation and lactation 6 weeks (analysis of dams)	Drinking water	ICR Mice (*Mus musculus*)	Shannon diversity index: no significant variation	No significant variation of *Bacteroidetes, Proteobacteria, Firmicutes* *↑ Actinobacteria* *↑ Epsilonbacteraeota*	14 significantly different
Li et al., Chemosphere. 2020 [93]	Polyethylene 10–150 μm	2–20-200 μg/g feed ~ 0.0004, 0.004 and 0.04 μg/kg bw/day*	5 weeks	Feed	C57BL/6 Mice (*Mus musculus*)	Shannon diversity index: ↑ in the 200 μg/g MP (p < 0.05)	↑ *Firmicutes* (20–200 μg/g), *↑Melainabacteria* (3 dosages) *↓ Bacteroidetes* (20–200)	*↑ Staphylococcus* *↓ Parabacteroides* (3 dosages)
Deng et al., Environment International 2020 [94]	Polyethylene 45–53 μm	100 mg/kg/day 5.25 10^4^ particles/day	30 days	Gavage	CD-1 mice (*Mus musculus*)	Shannon diversity index: no significant variation	*↑ Actinobacteria*	↑ *Lactobacillus* ↑ *Adlercreutzia* *↑ Butyricimonas* *↑ Parabacteroides*

In the juvenile guppy, exposure to PS microspheres (32–40 μm,100 and 1000 μg/L, for 28 days) induced dysbiosis, with a greater relative abundance of *Proteobacteria* and a lower relative abundance of *Actinobacteria* [80]. In larval zebrafish, exposure to PS microplastics (5 and 50 μm, 1000 μg/L, for 7 days) induced a decrease in gut microbiota richness and produced significant variations in the genus-level abundance [100]. In the large yellow croaker fish, 14 days of exposure to PS nanoplastics (100 nm, 5.5 × 10^− 12^ mg/L) enhanced the relative abundance of the *Firmicutes* and the *Bacteroidetes*, and diminished the abundance of the *Proteobacteria* [82]. In the medaka, exposure to PS particles (50 nm and 45 μm, 2.5 μg/mL, for 14 days) enhanced the abundance of *Bacteroidetes* phylum. At the genus level, PS nano- and microparticles decreased the abundance of *Vicingus* and *Shewanella,* and increased the abundance of *Lewinella*, *Pseudomonas*, *Thalassospira*, *Parahaliea* [83]. In the zebrafish, after 14-day exposure to high-dose of PS (1000 mg/L), the abundance of γ-*Proteobacteria* decreased significantly and the abundance of *Firmicutes* increased for both microbead size (0.5 and 50 μm) [84]. Another zebrafish study analyzed the effects of lower-dose exposure to PS microspheres (5 μm, 50 and 500 μg/L, for 21 days); it showed a decrease of bacterial diversity, an increase of the *Fusobacteria* abundance and a decrease of the *Proteobacteria* abundance [85]. The effects of PS fibres have also been assessed in the zebrafish: 21 days of exposure to 10 μg/L of PS fibres (20–100 μm) induced a diminution of bacterial diversity and variations in specific bacterial phyla (an enhancement in the *Proteobacteria* and a diminution in the *Actinobacteria*) [59].

Two studies in the mouse found a large number of significant modifications in the bacterial phyla composition after chronic exposure to PS microspheres (5 μm, 1000 μg/L, for 5 or 6 weeks) [60, 91]. The relative abundance of the α-*Proteobacteria* phylum was decreased by microplastic exposure in both studies, and the relative abundance of *Actinobacteria* and *Firmicutes* phyla were also reduced in the study by Lu et al. In contrast, the modifications in bacterial composition observed by Luo et al. were different: an increase in the abundance *Actinobacteria* but no significant variations in the *Proteobacteria* and *Firmicutes*. However, it should be noted that although Luo et al. used a very similar exposure protocol (5 μm PS beads, 1000 μg/L, for around 6 weeks), they exposed mice during gestation and lactation [101].

Two studies focused on PE microplastics. Li et al. (2020) observed that mice exposure to PE microplastics (10–150 μm, 2, 20 and 200 μg/g of food for 5 weeks) induced an increase in the abundance of the *Firmicutes* and *Melainabacteria* phyla and the *Staphylococcus* genus, and a decrease in the abundance of the *Bacteroidetes* phylum and the *Parabacteroides* genus [93]. Deng et al. (2020) observed that mice exposure to PE microplastics (45–53 μm, 100 mg/kg/day by gavage, for 30 days) increased the abundance of the *Actinobacteria* phylum and the abundance of *Lactobacillus*, *Adlercreutzia*, *Butyricimonas* and *Parabacteroides* genera [94].

In conclusion, all the reports aiming to study the intestinal microbiota in microplastic-exposed animals have observed dysbiosis. Even though the precise features of this dysbiosis vary from one context to another, the observed variations in microflora diversity and composition are likely to cause functional impairments of the immune system.

## IMMUNOTOXIC effects of ingested NANO- and MICROPLASTICS

The intestinal immune system interacts constantly with non-pathogenic commensal organisms and innocuous food antigens that must be tolerated immunologically. At the same time, the intestinal immune system must retain the ability to respond rapidly to infectious threats and toxins. This delicate task relies on several mechanisms involving myeloid cells, innate lymphoid cells, and T cells that reside in the intestinal lamina propria and the draining mesenteric lymph node. These immune cells circuits are critical components of the immune system. Even though the immunotoxicity of plastics has not been studied directly on the intestinal immune system, in vivo evidence of immunotoxicity of nano- and microplastics suggests that immune cells, including those of intestinal immune system, could be target for plastic-induced damage. Indeed, studies conducted mainly in invertebrates (Table 3) but also in vertebrates (Table 4) have demonstrated that their immune system is compromised by exposure to nano- and microplastics.

**Table 3:** Overview of in vivo studies of the immunotoxic effects of nano- and microplastics in invertebrates

Reference	Nano-micro plastics	Dosage	Duration of exposure	Route of exposure	Species	Observed immunotoxic effects
**Nanoplastics**						
Sadler et al., Environ. Pollut. 2019 [102]	Carboxylate- modified polystyrene beads 500 nm	1.25 ± 0.205 particles/L, or 85.6 ± 14.0 mg/L	1 year	Tank water	Cladoceran (*Daphnia magna)*	↑ **Hemocyte counts**
Brandts et al., Sci.Total Environ. 2018 [77]	Polystyrene ~ 110 nm	0.005–0.05-0.5-5-50 mg/L	96 h	Tank water	Mussel *(Mytiulus galloprovincialis)*	**Hemolymph** ↓total antioxidant capacity (5 mg/L) ↑ DNA damage (all dosages)
Auguste et al., Front.Immunol 2020 [103]	Amino-modified nanopolystyrene 50 nm	10 μg/L	24 h	Tank water	Mussel *(Mytilus. galloprovincialis)*	**Hemocytes** *One exposure:* ↓ mitochondrial membrane potential (MMP), ↑ lysosomal acidification ↓ lysosomal membrane stability ↑ lysozyme release No changes in total hemocyte count, subpopulations, phagocytic activity and ROS production ↓ transcription of PCNA and p53 No change in hemolymph bactericidal activity *Two exposures with 72 h resting period between*: normal hemocyte lysosomal stability, MMP, and lysozyme activity ↓ lysosomal membrane destabilization ↓ fully mature phagocytes ↑ bactericidal activity ↑ transcription of immune-related genes
Auguste et al. Marine Environmental Research 2020 [104]	Amino-modified nanopolystyrene 50 nm	10 μg/L	96 h	Tank water	Mussel *(Mytilus galloprovincialis)*	**Hemolymph** ↓ phagocytosis, ↑ ROS and lysozyme activity ↓ NO production. Hemolymph microbiota composition shift
**Microplastics**						
Paul-Pont et al., Environmental Pollution 2016 [76]	Polystyrene microbeads (2 and 6 μm)	32 μg/L	7 days	Supplied with *Chaetoceros mueller* algae as a food source	Mussel (*Mytilus spp*)	↑ hemocytes mortality and ROS production
Liu et al., Sci. Total Environ.,2019 [99]	Polystyrene 5 μm	0.04–0.4-4-40 mg/L	7, 14, and 21 days	Tank water	Crab *(Eriocheir Sinensis)*	**Immune parameters in the hemolymph** ***Hemocyanin content*** After 7 days: ↑ at 0.04 mg/L After 14 days: ↑ at 0.04 and 0.4 mg/L After 21 days: ↓at all dosages ***Acid phosphatase activity*** After 7 days: ↑ at 4 and 40 mg/L After 14 days: ↑ at 0.04 and 0.4 mg/L, ↓ 4 and 40 mg/L After 21 days: ↑ at 0.04 mg/L, ↓ 4 and 40 mg/L ***Alkaline phosphatase activity*** After 7 days: ↑ at 0.04 mg/L, ↓ 4 and 40 mg/L After 14 days: ↑ at 0.04 mg/L After 21 days: ↓ at all dosages ***Lysozyme activity*** After 7 days: ↓ at 40 mg/L After 14 days: ↓ at 0.4 and 4 mg/L After 21 days: ↓ at 4 et 40 mg/L ***Phenoloxidase activity*** After 7 days: ↑ at 0.04, 0.4, 4 mg/L, ↓ 40 mg/L After 14 days: ↓ at 0.4, 4, and 40 mg/L After 21 days: ↓ at all dosages **Expression of immune-related genes in the hemocytes** Hemocyanin and lysozyme: dose dependent ↓ Caspase: ↑ 0.04 and 4 mg/L, ↓ at 40 mg/L MyD88: ↑ at all dosages
Murano et al. Environmental Pollution 2020 [105]	Polystyrene microbeads (10 and 45 μm)	10 particles /mL	24 h 48 h 72 h	Tank water	Mediterranean sea urchin *(Paracentrotus lividus)*	↑ total number of immune cells ↑ ratio between red and white amoebocyte (at 3 times for 10 μm beads and only at 48 and 72 h for 45 μm beads) ↑ intracellular levels of reactive oxygen and nitrogen species (at 24 h only for both 10 and 45 μm beads) ↑ total antioxidant capacity (at 72 h for 10 μm beads)
Revel et al., Environ Sci Pollut Res Int. 2020 [106]	Polyethylene and Polypropylene 0.4–400 μm	10–100 μg/L	10 days	Soil	Ragworm (*Hediste diversicolor)*	**Coelomocytes** No variation of phagocytosis activity, phenoloxydase, and acid phosphatase
Revel et al., Mar. Pollut. Bull. 2020 [107]	Polyethylene and Polypropylene fragments < 400 μm	0.008–10-100 μg of particles/L	10 days	Tank water	Pacific oyster *(Crassostrea gigas)*	**Hemolymph** No variation of ROS production, acid phosphatase activity, and DNA damage
Green et al., Environ. Pollut. 2019 [108]	High density Polyethylene (HDPE) 0.48–316 μm Polylactic acid (PLA) 0.6–363 μm	HDPE 845 particles/L PLA 1296 particles/L	52 days 2 h/day	MP-dosed microalgae *Isochrysis galbana*	Blue mussel *(Mytilus edulis)*	**Hemolymph proteome** *HDPE group* Dysregulation of 6 protein involved in immune response ↑ three complement C1q domain-containing (C1qDC) proteins (FR715598.1; FR715581; HE609753.1), and fibrinogen-related protein (OPL33687.1) ↓ macrophage migration inhibitory factor (HE609105.1), Microfibril-Associated Glyco 4 (OPL32613.1) *PLA group* dysregulation of 3 protein involved in immune response ↑ C1Q Domain Containing 1Q19 (FR715598.1) and Fibrinogen-Related (OPL33687.1) ↓ Microfibril-Associated Glyco 4 (OPL32613.1)
Revel et al. Frontiers in Environmental Science 2019 [78]	Commercial polyethylene and polystyrene mixture (< 400 μm)	0.008, 10, 100 μg/L	10 days	Tank water	Mussel (*Mytilus spp.)*	**Hemolymph** No variation hemocyte count ↑ acide phosphatase activity (0.008 and 10 μg/L) ↑ DNA damage (10 and 100 μg/L)
**Both nanoplastics and microplastics**						
Shi et al., J. Hazard. Mater. 2020 [109]	Polystyrene beads 500 nm (NP) and 30 μm (MP)	0.29 mg/L	14 days	Tank water	Bivalve mollusk *(Tegillarca granosa)*	**Hemocytes** ↓ total hemocytes count ↓ phagocytosis ↓ viability (NP only) ↑ ROS content ↑ Caspase 3 activity ↑ malondialdehyde content ↓ ATP content (NP only) ↓ pyruvate kinase activity ↑ GABA content
Tang et al., Environ. Pollut. 2020 [110]	Polystyrene 500 nm (NP) and 30 μm (MP) and	1 mg/L	4 days	Tank water	Bivalve mollusk *(Tegillarca granosa)*	**Hemocytes** ↓ hemocytes count, basophils count, phagocytosis ↓ lysozyme (NP only) ↓ TLR4 (NP only), TRAF6, IKKα, NFκB gene expression ↑ Bcl2 (NP only), Caspase 3, Calmodulin gene expression

**Table 4:** Overview of in vivo studies of the immunotoxic effects of nano- and microplastics in vertebrates. *This has been calculated by the authors based on Bachmanov AA et al. *Behav Genet* 2002 [73]

Reference	Nano-microplastics	Dosage	Duration of exposure	Route of exposure	Species	Observed immunotoxic effects
**Nanoplastics**						
Greven et al., Environ. Toxicol. Chem. 2016 [111]	Polystyrene 41 nm	0.025–0.05-0.1- 0.2 μg/μL	2 h	Tank water	Fathead minnows *(Pimephales promelas)*	**Neutrophil function in vitro assays** Dose dependent ↑myeloperoxidase activity and neutrophil extracellular trap release
Brandts et al., Genomics. 2018 [112]	Polymethylmethacrylate ~ 45 nm	0.02–0.2-2-20 mg/L	96 h	Tank water	European sea bass (*Dicentrarchus Labrax)*	Plasma ↓ esterase activity (biomarker of oxidative stress) (0.02 and 0.2 mg/L)
**Microplastics**						
Greven et al., Environ. Toxicol. Chem. 2016 [111]	Polycarbonate 158.7 nm	0.025–0.05-0.1- 0.2 μg/μL	2 h	Tank water	Fathead minnows *(Pimephales promelas)*	**Neutrophil function in vitro assays** Dose dependent ↑ myeloperoxidase activity, neutrophil extracellular trap release, and oxidative burst
Asmoniate et al., Environ. Sci.Technol.2018 [79]	Polystyrene 100–400 μm	10 mg /fish/day	4 weeks	Food	Rainbow trout *(Oncorhynchus mykiss)*	No variation of immune parameters: serum lysosyme activity blood immune cells counts
Banaee et al., Chemosphere 2019 [113]	Isolated from body scrub (likely polyethylene) No data on size	250 and 500 μg/L	30 days	Tank water	Common carp *(Cyprinus carpio)*	Plasma ↓total immunoglobulin ↓alternative complement activity ↓ complement C3 ↓complement C4 ↓lysozyme activity ↓acetylcholinesterase activity ↓γ-glutamyl-transferase activity ↑ lactate deshydrogenase activity ↑alkaline phosphatase activity
Espinosa et al., Fish Shellfish Immunol. 2017 [114]	Polyvinyl chloride 40–150 μm	100–500 mg/kg of food	15, 30 days	Food	Gilthead seabream *(Sparus aurata)*	↑ head-kidney leucocyte phagocytic capacity (15 days only)
Espinosa et al., Environ. Pollut. 2018 [115]	Polyvinyl chloride - Polyethylene 40–150 μm	1–10-100 mg/mL	1 h 24 h	Tank water	European sea bass (*Dicentrarchus Labrax)* Gilthead seabream *(Sparus aurata)*	**Head-kidney leucocytes** *European sea bass* PVC: ↓phagocytic capacity (all dosages, 1 h and 24 h) PE: ↑ respiratory burst (100 mg/mL, 24 h) *Gilthead seabream* PVC: ↓phagocytic ability, ↑ respiratory burst (100 mg/mL, 24 h) PVC and PE: ↑ *Nrf2* expression (100 mg/mL, 1 h)
Espinosa et al., Fish Shellfish Immunol. 2019 [88]	Polyvinyl chloride - Polyethylene 40–150 μm	100–500 mg/kg of food	3 weeks	Food	European sea bass (*Dicentrarchus Labrax)*	**Cellular innate immune parameters in head kidney leucocytes** PVC: ↑ phagocytic capacity at both dosages ↑ respiratory burst activity at 100 mg/kg PE: ↑ respiratory burst activity at both dosages
Limonta et al., Sci rep 2019 [90]	Irregularly shaped high density polyethylene and polystyrene particles	100 and 1000 μg/L	20 days	Food	Zebrafish (*Danio rerio)*	↓ liver leukotriene B4 receptor (*ltb4r*) and interferon induced transmembrane protein 1 (*ifitm1*) expression
Li et al., Chemosphere. 2020 [93]	Polyethylene 10–150 μm	2–20-200 μg/g ~ 0.0004, 0.004 and 0.04 μg/kg bw/day*	5 weeks	Food	C57BL/6 mice *(Mus musculus)*	At 20 and 200 μg/g: serum:↑ IL1α, ↓G-CSF spleen:↓Treg cells, ↑Th17 cells
Park et al., Toxicology Letters 2020 [116]	Polyethylene irregular micropsheres 16.9 ± 1.9 μm	0.125, 0.5 and 2 mg/day/mouse ~ 5, 20 an 80 μg/kg bw/day*	90 days	Gavage	ICR mice *(Mus musculus)*	In dams: ↑ blood neutrophils ↑ blood IgA levels In dams and offspring: alteration of spleen lymphocytes

### In vivo immunotoxicity of nano- and microplastics in invertebrates

Several studies of invertebrates have linked PS exposure to disruption of the immune system. Exposure of cladoceran *Daphnia magna* to carboxylate-modified PS nanoparticles (500 nm, 85 mg/L, for 1 year) was associated with higher hemocyte counts [102]. In mussel hemolymph, exposure of PS particles (110 nm, 5 mg/L, for 96 h) decreased total antioxidant capacity and gave rise to DNA damage [77]. Exposure of mussels to amino-modified PS nanoparticles (50 nm, 10 μg/L) induced changes in hemocytes, depending on the duration of exposure. After a 24 h exposure, the hemocytes presented mitochondrial and lysosomal disturbances [103]. After two 24 h periods of exposure 72 h apart, levels of bactericidal activity and immune-related gene transcription were found to be elevated. After 96 h of exposure, hemolymph phagocytosis, levels of oxidative stress, and the microbiota were modified [104].

Several research groups have explored the effects of PS on the microscale. In mussels, hemocytes mortality and reactive oxygen species production were increased by exposure to PS microbeads (2 and 6 μm, 32 μg/L, for 7 days) [76]. In crab hemolymph, the hemocyanin content and the levels of activity of several enzymes related to the immune system (acid phosphatase, alkaline phosphatase, lysozyme and phenoloxidase) were significantly modified by PS exposure (5 μm, 0.04 to 40 mg/mL, for 7, 14 or 21 days), although the direction of change (i.e. an increase or a decrease) varied with the duration or dose level of PS exposure [99]. In Mediterranean sea urchin, exposure to PS microbeads (10 μm and 4 5 μm, 10 particles/mL, for 24 h) increased the total coelomocyte count and the intracellular levels of reactive oxygen and nitrogen species, indicating a stress-related impact on these circulating immune cells [105]. In the bivalve mollusk *Tegillarca granosa*, two studies have shown that PS exposure (500 nm and 30 μm, 0.29 and 1 mg/L, during 14 and 4 days, respectively) leads to several disturbances in hemocytes, with notably a decrease in the cell count and in phagocytosis activity, and numerous variations in immune parameters related to oxidative stress, apoptosis, and the inflammatory response [109, 110]. In both studies, PS nanoparticles caused more damage than PS microparticles did.

Other microplastics have been studied individually. Polyethylene and PP fragments (< 400 μm, 0.008 to 100 μg/L, for 10 days) did not show significant immune damage in ragworm and oyster [106, 107]. Specific dysregulation of proteins involved in immune response were observed in hemolymph of mussels exposed to PE and polylactic acid microplastics (845 and 1296 particles/L respectively, 2 h/day, for 52 days) [108].

Lastly, in *Mytilus spp*, exposure to a mixture of PE and PS microbeads (< 400 μm, 10 μg/L, for 10 days) enhanced hemolymph acid phosphatase activity and DNA damage [78].

### In vivo immunotoxicity of nano- and microplastics in vertebrates

Two studies have assessed the in vivo immunotoxicity of nanoplastics in fishes. In vitro neutrophil function assays showed dose-dependent increases in myeloperoxidase activity and neutrophil extracellular trap release in fathead minnows *Pimephales promelas* exposed to PS nanoparticles (41 nm, 0.025 to 0.2 μg/μL) [111]. Acute exposure to PMMA nanoparticles (45 nm, 0.02 and 0.2 mg/L, for 96 h) diminished the level of oxidative stress in the plasma [112].

Several studies have explored the in vivo toxicity of micro scale plastics. Polycarbonate microplastics (159 nm, 0.025 to 0.2 μg/ml, 2 h) dose-dependently disturbed neutrophil function in fathead minnows [111]. In contrast, no significant immunotoxicity was observed in trout exposed for 4 weeks to PS microbeads (100–400 μm, 10 mg/fish/day) [79]. Exposure to PE microparticles (250 and 500 μg/L, for 30 days) impaired the complement system and the levels of activity of immunity-related enzymes in the plasma of carp [113]. Espinosa et al. showed in fishes that exposure to PVC microplastics (40–150 μm, 1 to 500 mg/kg, for between 1 h and 3 weeks) disturbed phagocytic capacity and increased the head-kidney leukocytes’ respiratory burst [88, 114, 115]. Polyethylene also enhanced the head-kidney leukocytes’ respiratory burst in fishes, and dysregulated major immune response proteins in the hemolymph of mussels [88, 115]. In zebrafish, exposure to high-density PE and PS particles (100 and 1000 μg/L, for 20 days) decreased the liver transcript levels of 2 immune genes leukotriene B4 receptor (*ltb4r*) and interferon induced transmembrane protein (*ifitm1*) [90].

In mice, exposure to PE microplastics (10–150 μm, 20 and 200 μg/g, for 5 weeks) modified the serum levels of IL1α and granulocyte colony-stimulating factor G-CSF, decreased the regulatory T cell count, and increased the proportion of Th17 cells in splenocytes [93]. The crossgenerational effects of PE exposure (7 μm, 0.125 to 2 mg/day/mouse, for 90 days) have been also studied in the mouse: blood neutrophil counts and IgA levels were elevated in dams, and spleen lymphocytes were altered in both dams and offspring [116].

Lastly, Mancia et al. studied the small-spotted catshark (*Scyliorhinus canicula*) in the Mediterranean Sea: the presence of macroplastics in the gastrointestinal tract was associated with significant upregulation of the expression of T cell receptors beta and delta (TCRβ and TCRδ) and immunoglobulin M (IgM) in the spleen [117].

The many alterations observed in these studies not only demonstrate that the immune system is altered by plastics but also highlight the need for more immunotoxicity studies of species more closely related to the human.

## MICROPLASTICS as carriers of intestinal toxics and pathogens

Microplastics may contain additives on average (4% w/w, on average) and can adsorb contaminants [63, 118]. Both additives and contaminants can be of organic as well of inorganic nature. The main plastic additives and adsorbed contaminants for which data are available are the phthalates, bisphenol A, polybrominated diphenyl ethers, polycyclic aromatic hydrocarbons (PAHs) and polychlorinated biphenyls (PCBs). Concentrations of up to 2750 ng/g of PCB [119] and 24,000 ng/g of PAHs [120] have been found in microplastics deposited on beaches.

The presence of additives and contaminants in microplastics raises concerns about the latter’s ability to accentuate the bioaccumulation of some of them which could be intestinal toxics. Indeed, the bioaccumulation of oxytetracycline and florfenicol, two frequently detected veterinary antibiotics, in edible bivalves (clams) was found to be aggravated by co-exposure to microplastics (PS particles, 500 nm, 0.26 mg/L) [121]. It has been shown that microplastics carry several known intestinal toxics, such as polybrominated diphenyl ethers [122], cadmium [113, 123] and triclosan [124]. In mice, microplastics adsorb phthalate esters and can transport them into the gut, where they accumulate [94].

Secondly, the presence of additives and contaminants in or on microplastics raises concerns about the possible accentuation of the pollutants’ toxicity. Indeed, microplastics aggravated the immunotoxicity of bisphenol A and petroleum hydrocarbons in blood clams [125, 126], and the immunotoxicity of cadmium in fish [113]. Polyethylene microplastics increased the toxicity of the pesticide chlorpyrifos in the marine copepod *Acartia tonsa* [127]. In mice, gut inflammation induced by exposure to organophosphorus flame retardants was aggravated by PE or PS co-exposure (0.5–1 μm beads, 2 mg/L of drinking water, for 90 days) [61]. Exposure of mice to di (2-ethylhexyl) phthalate (DEHP)-contaminated microplastics for 30 days worsened histological signs of intestinal inflammation, and impairments in intestinal permeability (as revealed by the serum D-lactate level and serum diamine oxidase activity) [94]. Although few data on the intestine per se are available, the above-cited studies demonstrate that microplastics can add to or synergize the adverse effects of the toxics that they contain or have absorbed.

Furthermore, microplastics house distinct communities of microbes, that can form fully developed surface biofilms [128, 129]. Plastic debris supports the growth of specific bacterial consortia, including bacterial pathogens. For example, *Vibrio spp*. and *Escherichia coli* have been repeatedly found in microplastic biofilms [128]. These biofilms differ in their microbial composition, relative to biofilms formed on natural substrates [130]. For example, recent analyses of biofilms on microplastics and on two natural substrates (rock and leaf) led to the detection of two opportunistic human pathogens (*Pseudomonas monteilii* and *Pseudomonas mendocina*) in the microplastic biofilm only [130]. Therefore, microplastics may serve as vectors for pathogens. Furthermore, the complex microbial consortia in microplastic biofilms may promote horizontal gene transfer between phylogenetically distinct microbes more rapidly than in free-living microbes. Hence, microplastics could also serve as ‘hotspots’ for the development and dissemination of various drug-resistant human pathogens via co-selection mechanisms [131, 132]. In accordance with this concept, it has been shown in mussels that the gut dysbiosis observed after exposure to PE microplastics is associated with an increase of the relative abundance of some potential human pathogens [133]. Exposure to microplastic biofilms is likely to trigger changes in the gut microbiota and activation of the immune system, although this field has not yet been explored.

## Conclusions

The gut epithelium encounters a broad range of plastics. Faced with this complexity, we still have too few data on the amounts and features of ingested plastics. Analytical limitations on the detection of particles sizing few μm mean that the environmental exposure data relates to micro-size plastics only. Moreover, these exposure data are subject to debate because of the limited number of studies of food products, poor data quality (due to contamination, for example), and the absence of data on small particles [36, 134]. Furthermore, interstudy comparisons are not valid because of the lack of standardized technical methods for collection and analysis. Today’s available data give us a few clues about the plastic pollutants in drinking water and in a small number of food products. Larger studies of plastics in the general diets, (i.e. providing a realistic estimate of overall oral contamination by plastics) are still lacking. With regards to this challenge, we suggest that research should initially focus on the microplastics present in human stools. This strategy is particularly useful for identifying the plastics that exert their harmful effects via direct contact with the intestinal mucosa. Furthermore, it is essential to study the nano- and microplastics that cross the intestinal barrier; even small quantities of translocated plastic may be as dangerous as or even more dangerous than plastics excreted in the stools. Lastly, in view of the recent literature on the effects of particulate matter in the atmosphere, the impact of airborne microplastics on the intestine should also be assessed [135–137].

To the best of our knowledge, the intestinal and immunotoxic effects of nanoplastic ingestion by mammals have never been studied. However, ingestion of non-plastic nanoparticles is known to have many harmful effects. For example, ingestion of TiO_2_ nanoparticles by mammals impairs intestinal and systemic immune homeostasis and induces variations in the gut microbiota and gut-associated metabolism [138, 139]. One can therefore reasonably hypothesize that contamination of the diet by some nanoplastics is likely to harm intestinal and immune systems; this topic requires more attention. Furthermore, the microplastics most frequently studied for their in vivo effects are PS and (to a lesser extent) PP: more efforts are required for PP and PET, the two most abundant microplastics in human feces.

Despite the lack of data on plastic levels in the human diet, it is clear that plastics contaminate water and the food chain; the toxicological effects of certain types and shapes of microplastics are now starting to be assessed. The current data are insufficient and do not allow robust scientific conclusions to be drawn for humans in general and intestinal health in particular. In this respect, the lack of reliable data on human dietary exposure means that relevant doses have yet to be defined. Furthermore, the effects of chronic exposure to microplastics appear to be very variable and dependent on the latter’s type and shape. It is also very likely that the effects of a cocktail of microplastics (as encountered in real life) are different from those of individual components - further complicating the problem. Likewise, the biological relevance of the research results – notably concerning the impact on the immune system - would be increased by studies of nano- and microplastics bearing biofilms. However, most of the literature studies suggest that nano- and microplastics have several effects on intestine: the disturbance of intestinal homeostasis, alterations in gut permeability, and changes in the recruitment of immune cells or in levels of cytokine secretion. The intestinal dysbiosis which has been observed following microplastic ingestion, sometimes differs from one study to another but reflects the deregulation of a crucial parameter for host defense, intestinal metabolism and inflammation. The immune system’s susceptibility to plastics constitutes an additional threat to health.

**Scheme 4:**
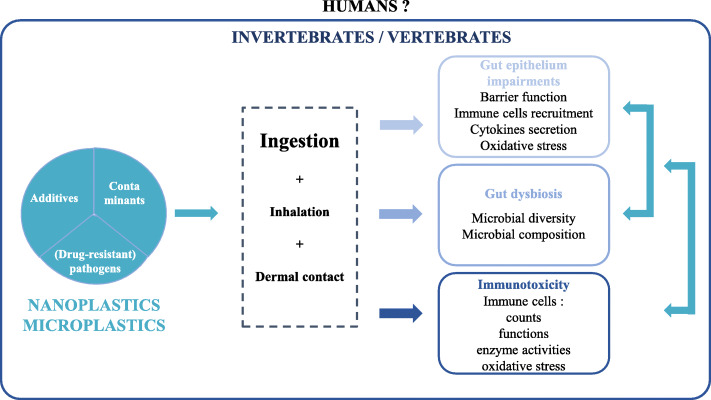
Overview of the potential effects of nano- and microplastic contamination on intestinal health and the immune response

In conclusion, a growing body of evidence shows that the omnipresence of plastics in our daily life is associated with chronic, evolving exposure to microplastics. Furthermore, many animal experiments suggest that the ingestion of microplastics disrupts essential intestinal functions, such as the gut barrier function and regulation of the gut microbiota (Scheme 4). Due to the multifunctional nature of the intestinal system, these plastic-associated disruptions may promote immune, inflammatory and metabolic disorders and therefore warrant further investigation.

## Data Availability

Not applicable.
